# Impact of the Reticular Stress and Unfolded Protein Response on the inflammatory response in endometrial stromal cells

**DOI:** 10.1038/s41598-018-29779-8

**Published:** 2018-08-16

**Authors:** E. Grasso, S. Gori, E. Soczewski, L. Fernández, L. Gallino, D. Vota, G. Martínez, M. Irigoyen, C. Ruhlmann, T. F. Lobo, G. Salamone, R. Mattar, S. Daher, C. Pérez Leirós, R. Ramhorst

**Affiliations:** 1 CONICET, Universidad de Buenos Aires. Instituto de Química Biológica de la Facultad de Ciencias Exactas y Naturales IQUIBICEN, Buenos Aires, Argentina; 2 Institute of Experimental Medicine IMEX-CONICET, National Academy of Sciences, Buenos Aires, Argentina; 3 Instituto de Fertilidad San Isidro, Buenos Aires, Argentina; 4 Department of Obstetrics, Universidade Federal de São Paulo, São Paulo, Brazil

## Abstract

During decidualization, endometrial stromal cells undergo reticular stress (RS) and unfolded protein response (UPR), allowing the endoplasmic reticulum-expansion and immunomodulators production. Physiological RS generates the activation of sensing proteins, inflammasome activation and mature-IL-1β secretion, associated with pro-implantatory effects. We focus on the impact of RS and UPR on decidualized cells and whether they induce a physiological sterile inflammatory response through IL-1β production. Human endometrial stromal cell line (HESC) after decidualization treatment with MPA + dibutyryl-cAMP (Dec) increased the expression of RS-sensors (ATF6, PERK and IRE1α) and UPR markers (sXBP1 and CHOP) in comparison with Non-dec cells. Then we found increased NLRP3 expression in Dec cells compared with Non-dec cells. In fact STF-083010 (an IRE1α inhibitor) prevented this increase. Downstream, increased levels of active caspase-1 on Dec cells were detected by FAM-Flica Caspase-1 associated with an increase in IL-1β production. Moreover, the treatment with STF-083010 decreased the invasion index observed in Dec cells, evaluated by an *in vitro* model of implantation. In endometrial biopsies from recurrent spontaneous abortion patients an increased expression of IRE1α was found in comparison with fertile women; while recurrent implantation failure samples showed a lower expression of sXBP1, TXNIP and NLRP3 than fertile women, suggesting that RS/UPR tenors might condition endometrial receptivity.

## Introduction

Embryo implantation in humans involves the generation of an inflammatory response associated with the invasion of the blastocyst into the decidua. The embryo has to break through the epithelial lining of the uterus to implant, damage the endometrial tissue to invade and replace the endothelium and vascular smooth muscle of the maternal blood vessels^1–3^. This inflammatory response is sterile and could be induced by endogenous damage ligands (DAMPs: Damage-associated molecular patterns) released during tissue remodeling; however, it is still unclear whether other processes are involved.

A singular feature of the reproductive cycle in humans is the ‘spontaneous’ decidualization of the endometrium in an embryo-independent manner. The decidualization program involves not only morphological alterations of the endometrial stromal cells, but it also involves changes in their secretome associated with the production of proinflammatory factors^4,5^. In this sense, the increase in the secretion of these mediators would be accompanied by a physiological reticular stress (RS) and unfolded protein response (UPR), which allow cells to expand their endoplasmic reticulum (ER) with the corresponding machinery for protein folding^6,7^.

The UPR is a sophisticated network of intracellular signaling pathways that have evolved to sustain an adequate folding and post-translational modifications of proteins. It is crucial for maintaining cellular homeostasis in case of misfolded or aggregated proteins occur. Under prolonged activation, it could trigger autophagy and later the terminal UPR that leads to cell death^8,9^. Three transmembrane sensors, the serine/threonine kinase inositol-requiring enzyme 1 α (IRE1α), PKR-like ER-localized eIF2a kinase (PERK) and the activating transcription factor 6 (ATF6) collectively determine the cellular response to RS signals and together elicit a program of gene expression designed to maintain cellular homeostasis. Numerous environmental conditions, both endogenous and exogenous, can disrupt the protein folding environment, causing the accumulation of misfolded and unfolded proteins in the ER lumen which trigger RS/UPR^10,11^.

IRE1α and PERK play critical roles in regulating the thioredoxin-interacting protein (TXNIP) expression. In the case of IRE1α, this regulation depends on its dual functions as a protein kinase and as an endoribonuclease. IRE1α kinase domain is autophosphorylated and induces the splicing of XBP-1 (X-box binding protein-1) to the spliced form known as sXBP1. Then, sXBP1 transactivates a subset of target genes that are involved in protein transport, protein folding, ER-associated protein degradation, protein translocation to the ER and protein secretion such as the C/EBP-homologous protein (CHOP)^8,12^.

The increment of TXNIP mRNA stability by IRE1α leads to elevated TXNIP protein levels that promote the formation of large multiprotein complexes called inflammasomes. NOD-like receptor (NLR) proteins are key components of inflammasomes, which facilitate Caspase-1 maturation and cytokines secretion in response to cellular danger^10,13–15^. Lerner *et al*. (2012) showed that treatment with STF-083010, a drug that inhibits IRE1α endoribonuclease function without affecting its kinase activity, reduces TXNIP expression and inflammasome activation in pancreatic β-cells^14–17^. Therefore, the IRE1α-TXNIP pathway activates the NLRP3 inflammasome, inducing Caspase-1 cleavage and interleukin 1β (IL-1β) secretion that, in the terminal UPR, promotes sterile inflammation in pancreatic β-cells^15^. So far, the induction of a sterile inflammatory pathway associated with the differentiation of endometrial stromal cells has not been studied.

In the present study, we evaluated the induction of the RS and UPR processes during the decidualization as an inductor of a sterile and physiological inflammatory response with NLRP3 inflammasome activation, Caspase-1 maturation and IL-1β production. Furthermore, we investigated if alterations in the RS/UPR levels and IL-1β production, either reduced or exacerbated levels, might precondition the endometrium from patients with recurrent implantation failures (RIF) or recurrent spontaneous abortions (RSA).

## Results

### The decidualization program is accompanied by an increase in the expression of RS sensors and activation of UPR pathway

**Figure 1:**
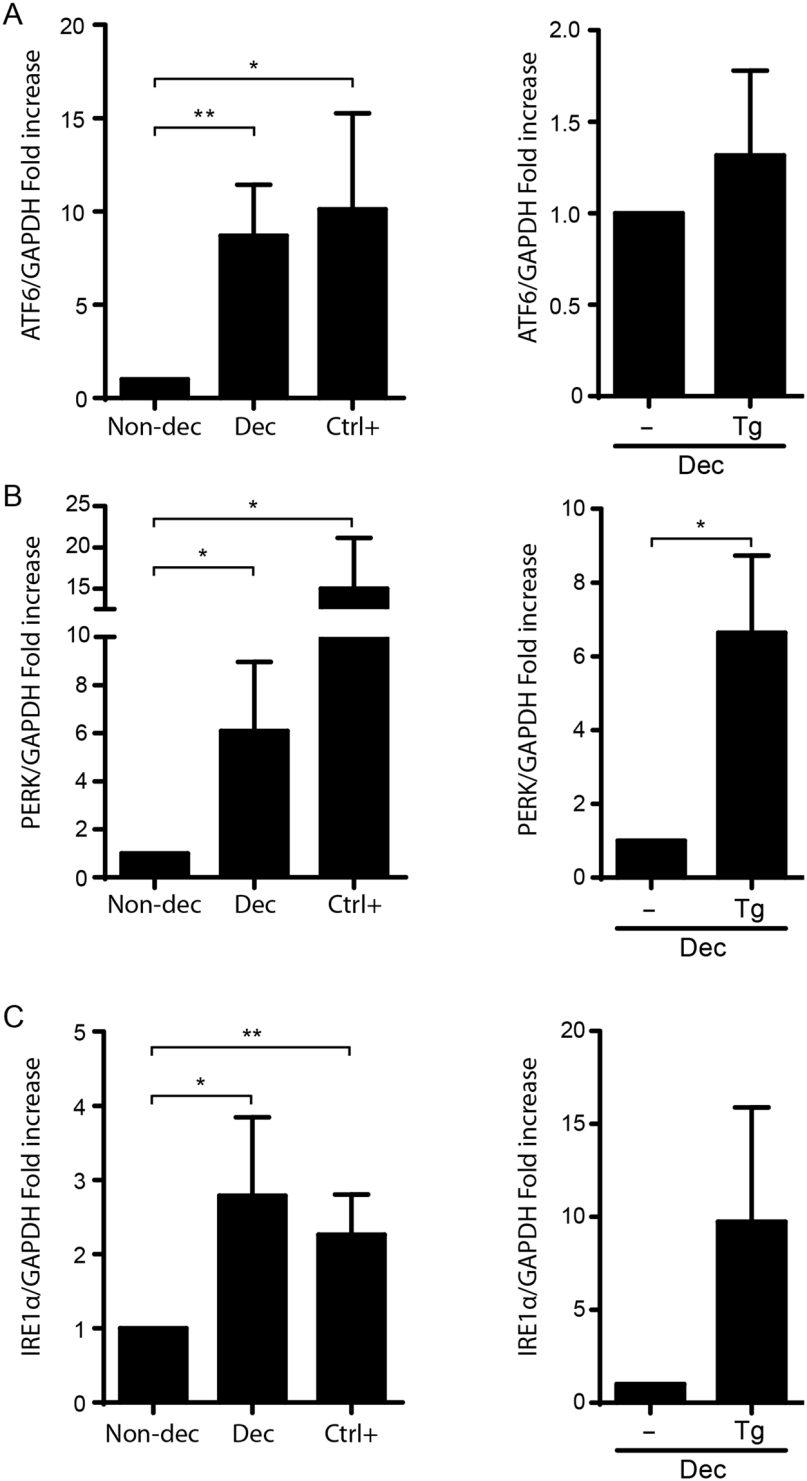
The decidualization program is accompanied by an increase in the expression of RS sensors. Non-decidualized cells (Non-dec) and decidualized cells (Dec) were evaluated for the expression of RS sensors: (**A**) IRE1α, (**B**) PERK and (**C**) ATF6. As RS positive control (Ctrl+; Tg) HESC cells were cultured in the presence of RS-inducer Thapsigargin (1 μg/ml for 4 h). Results for qPCR are expressed as fold increase in respect to Non-dec cells (left panel) or to Dec cells (right panel). Bars: Mean ± S.E.M. of at least 4 independent experiments. *p < 0.05, **p < 0.01, Paired Student’s t-test.

Decidualization of the endometrial stromal cells involves changes in their secretome that should be accompanied by a physiological RS. Therefore, first we evaluated the expression of the three RS sensors both in decidualized HESC cells by treatment with MPA (10^−7^ M) and dbcAMP (2, 5 mM) during 8 days (Dec) and in non-decidualized cells (Non-dec). Both cultures were performed also in the presence of Thapsigargin (Tg), an RS-inducer, showed in left (Ctrl+) and right (Tg) panels of Fig. 1A–C. This figure shows a significant increase of ATF6, PERK and IRE1α expression after decidualization process. Indeed, Tg-treated Non-dec HESC cells (Ctrl+) significantly increased the expression of the three sensors. As it is shown in each right panel from Fig. 1A–C, an interesting point is that decidualized HESC cells still have the ability to respond to a strong RS-inducer, suggesting that decidualization displays low or mild RS levels.

**Figure 2:**
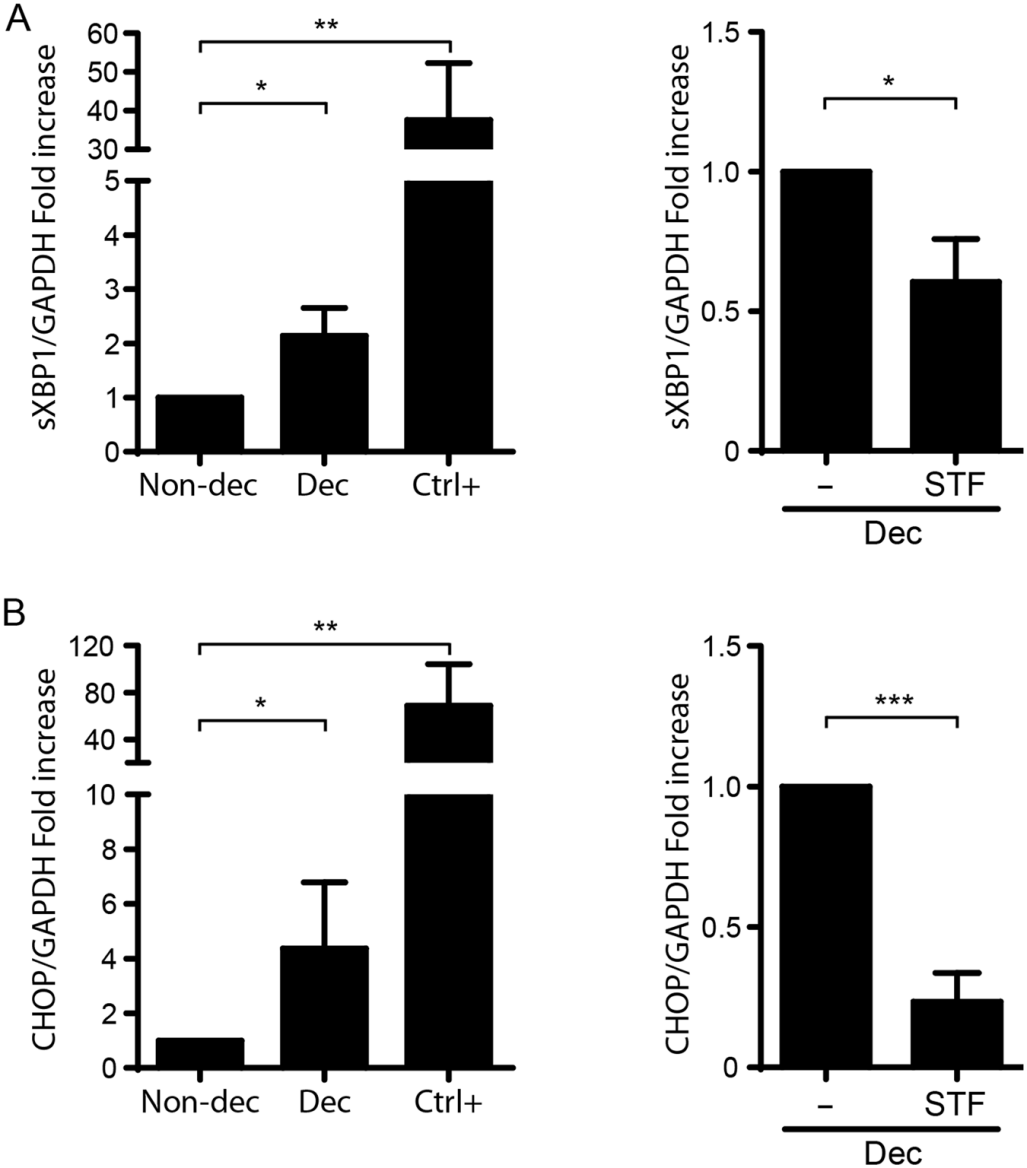
Decidualization stimulates the UPR pathway. Non-decidualized cells (Non-dec) and decidualized cells (Dec) were evaluated for the expression of UPR mediators as (**A**) the transcription factor sXBP1 and (**B**) the C/EBP-homologous protein CHOP. As a positive control (Ctrl+) HESC cells were also cultured in the presence of Tg (1 μg/ml for 4 h). Dec cells were also cultured in the presence of STF-083010 (an inhibitor of IRE1α, 10 μg/ml for 4 h). Results for qPCR are expressed as fold increase in respect to Non-dec cells (left panel) or to Dec cells (right panel). Bars: Mean ± S.E.M. of at least 5 independent experiments. *p < 0.05, **p < 0.01, Paired Student’s t-test.

Next, we evaluated the UPR pathway through the expression of the transcription factor activator sXBP1. The spliced form of XBP1 known as sXBP1 transactivates a subset of target genes that are involved into the UPR as the C/EBP-homologous protein (CHOP). Therefore, sXBP and CHOP expression were evaluated in Non-dec cells in the absence/presence of Tg (Ctrl+) as previously described, and after decidualization (Dec cells). As depicted in Fig. 2A,B, sXBP1 and CHOP expression significantly increased after decidualization. To determine if these modulations are specifically due to RS/UPR induction, we performed the same cultures in the presence of STF-083010, an RS-inhibitor of the IRE1α pathway. This inhibitor was able to prevent the increase in sXPB1 and CHOP expression (Fig. 2A,B, right panels). Taking together, the present results suggest that the decidualization process is accompanied by low or mild levels of RS and UPR.

### The decidualization program activates NLRP3 inflammasome inducing a sterile inflammatory response through IL-1β production

**Figure 3:**
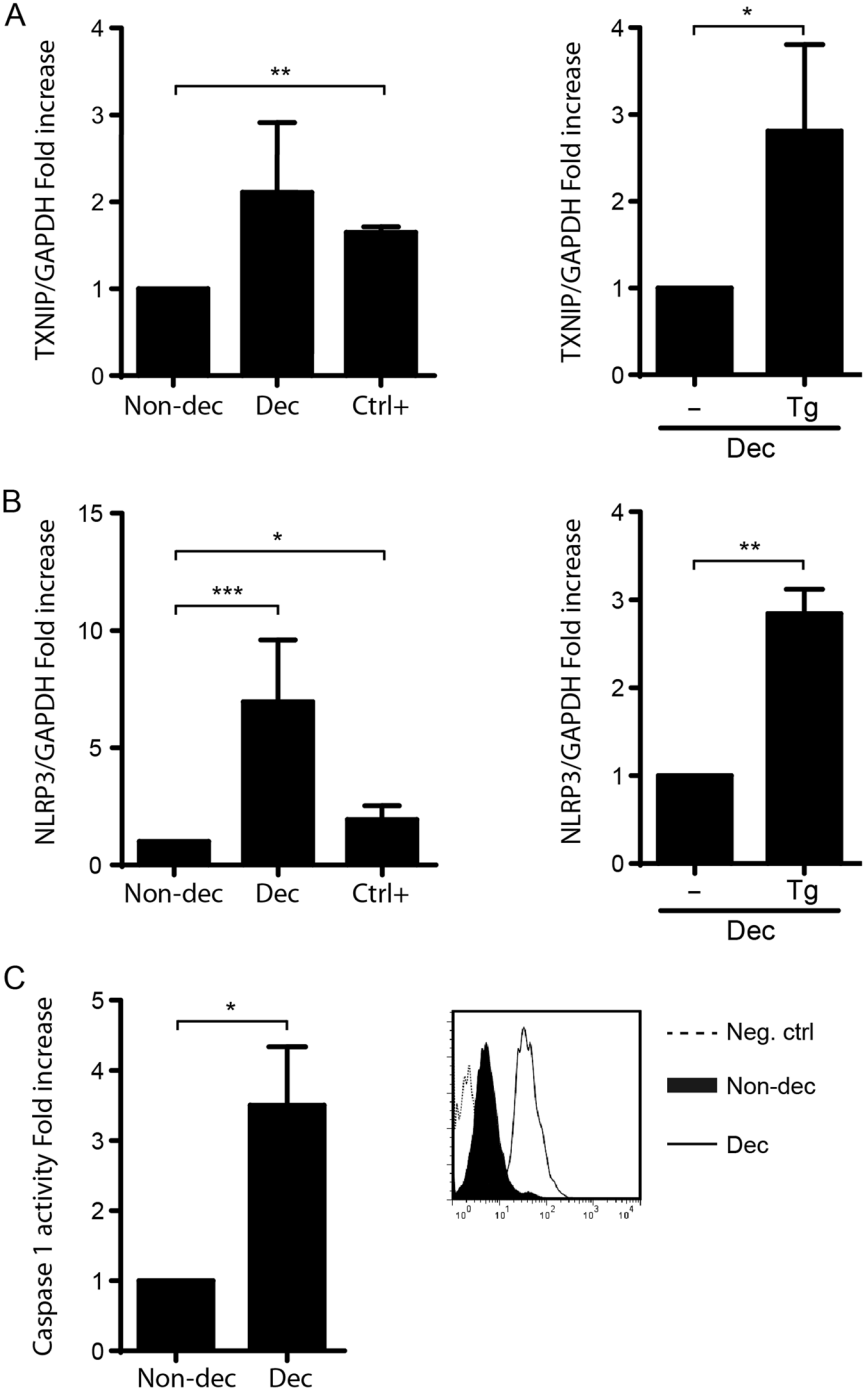
The decidualization program activates NLRP3 inflammasome which induces caspase-1 maturation. Non-decidualized cells (Non-dec) and decidualized cells (Dec) were evaluated for the expression of (**A**) TXNIP and (**B**) NLRP3. As RS positive control (Ctrl+) assays were performed in the presence of Tg (1 μg/ml for 4 h). Results for qPCR are expressed as fold increase in respect to Non-dec cells (left panel) or to Dec cells (right panel). Bars: Mean ± S.E.M. of at least 5 independent experiments. (**C**) Caspase-1 activity was evaluated by the fluorescent inhibitor probe FAM-Flica and analyzed by flow cytometry. Results were expressed as fold increase of the MFI to the Non-dec cells. The right panel shows a representative histogram obtained from 3 independent experiments. Cells without FLICA treatment were used as negative autofluorescence control. Bars: Mean ± S.E.M. *p < 0.05, **p < 0.01, ***p < 0.001 Paired Student’s t-test.

Since a link between the RS/UPR and the activation of a sterile inflammatory response in pancreatic β-cells was reported, and taking into consideration that human embryo implantation is accompanied by a physiological inflammatory response, we next evaluated if the decidualization program could induce the inflammasome activation. Therefore, we evaluated the expression of TXNIP, a link between UPR and inflammasome activation, and the expression of NLRP3, a key component of inflammasomes by RT-qPCR. As show Fig. 3A,B, decidualization significantly increases TXNIP and NLRP3 expression as well as Non-dec HESC cells treated with Tg (Ctrl+). In fact, HESC cells after decidualization still have the ability to respond to a strong RS-inducer, suggesting again that decidualization displays low or mild levels of RS/UPR that induce inflammasome activation, as it was also shown for the markers studied in Fig. 1.

Since the inflammasome regulates the activation of Caspase-1, we evaluated Caspase-1 activity using a fluorescent inhibitor probe FAM-Flica and flow cytometry analysis. Figure 3C shows that Dec cells significantly increase Caspase-1 activity, confirming that the multiprotein-complex has functional activity and could be induced by RS/UPR in Dec cells.

**Figure 4:**
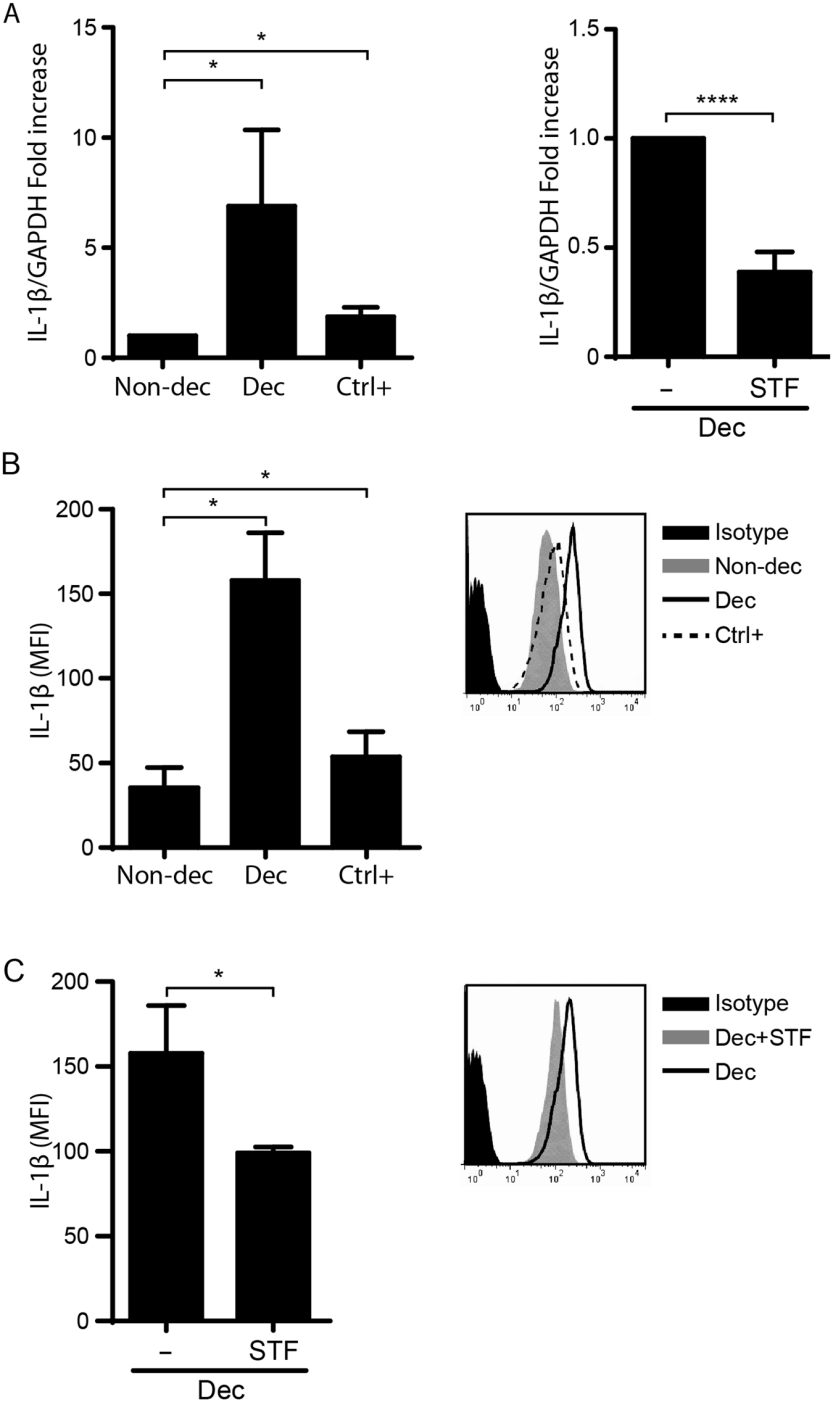
The decidualization program activates the inflammasome inducing IL-1β production. The production of IL-1β in HESC was evaluated by (**A**) IL-1β expression on Non-decidualized cells (Non-dec) and decidualized cells (Dec) by RT-qPCR and (**B**) active IL-1β protein levels by intracellular cytokine detection by flow cytometry analysis. As a positive control (Ctrl+) HESC cells were cultured in the presence of Tg (1 μg/ml for 4 h) **(A**, right panel and **B)**. In addition, Dec cells were cultured in the presence of IRE1α inhibitor, STF-083010 (10 μg/ml for 4 h) **(A**, left panel and **C)**. Results for RT-qPCR **(A)** are expressed as fold increase in respect to Non-dec cells (left panel) or to Dec cells (right panel). Flow cytometry results were expressed as the MFI of IL-1β and representative histograms are shown. Bars: Mean ± SEM of at least 5 independent experiments. *p < 0.05, ****p < 0.0001, Paired Student’s t-test.

Taking into account that the activation of Caspase-1 controls the maturation of two inflammatory cytokines (IL-1β and IL-18), and particularly that IL-1β also is a key pro-implantatory factor, we investigated the IL-1β production before and after decidualization by intracellular staining and flow cytometry analysis. Figure 4 shows that IL-1β expression (Fig. 4A) and production (Fig. 4B,C) significantly increased after the decidualization process as well as the Ctrl+. Moreover, STF-083010 treatment significantly decreased IL-1β expression and production in Dec cells, suggesting that the reduction of RS/UPR, particularly through IRE-1α pathway, is associated with a decrease in IL-1β production.

### RS and UPR prevention decrease trophoblast cell invasion in an *in vitro* model of embryo implantation

**Figure 5:**
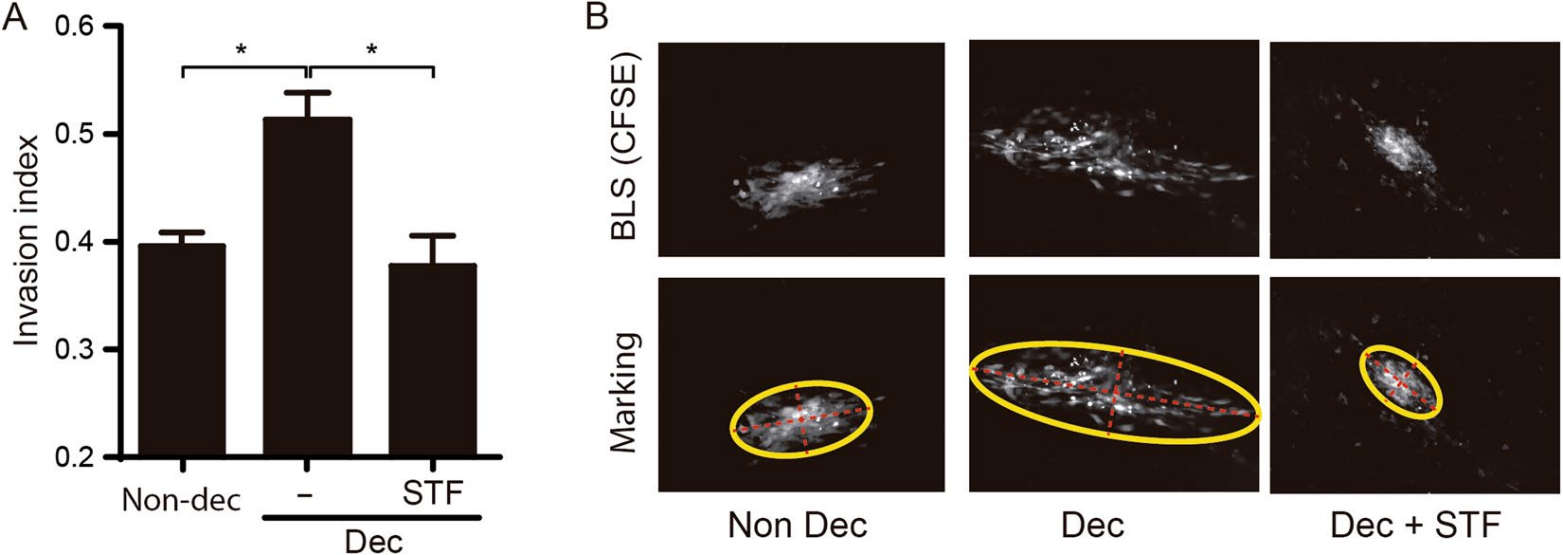
RS and UPR prevention decreases trophoblast cell invasion in an *in vitro* model of embryo implantation. Swan-71 cells were cultured on non-adherent plates for 24 to 48 h to form the blastocyst-like spheres (BLS). The BLS were morphologically selected, tagged with CFSE and seeded over non-decidualized or decidualized HESC cells monolayer. (**A**) The BLS invasion was followed during 48 h by fluorescence microscopy and morphologically analyzed (*p < 0,05 ANOVA Sidak posttest. Bars: Mean ± SEM from 6 independent experiments). (**B)** First row shows representative microscopy pictures of BLS tagged with CFSE invading the HESC monolayers under different treatment. Invasion index was calculated as “1-(minor_axis/mayor_axis)” of an ellipse surrounding the BLS. The second row, shows the same pictures with marks used to calculate the invasion index (ellipse in yellow, axis in red).

Since during decidualization, RS/UPR induces the activation of the inflammasome and the production of IL-1β, we wondered if the RS/UPR prevention could reduce the blastocyst implantation ability. To answer this question, we used an *in vitro* model of human embryo implantation that we have previously described in Grasso *et al*.^18^. Briefly, Swan-71 cells, a human first trimester trophoblast cell line, were cultured on non-adherent plates for 48 h to form blastocyst-like spheroids (BLS). The BLS were morphologically selected, tagged with CFSE and seeded over confluent monolayers of Dec or Non-dec cells. All co-cultures and each BLS were monitored by fluorescence microscopy and, after 48 h, the invasion index was obtained as described in the M&M section. Figure 5A shows that Dec cells allowed BLS invasion. However, if Dec cells were pre-treated with the IRE1α inhibitor, STF-083010, the BLS invasion was prevented. Figure 5B shows representative microphotographs of BLS, tagged with CFSE, that was invading the HESC monolayers under different treatments. The present results support that physiological RS/UPR not only induces a sterile inflammatory pathway in Dec cells, but also allows them to be receptive for blastocyst invasion evidenced in an *in vitro* model of embryo implantation.

### RS/UPR through IRE1α pathway and the induction of IL-1β are differentially expressed in endometrium from patients with recurrent spontaneous abortion and from recurrent implantation failures in comparison with fertile women

**Figure 6:**
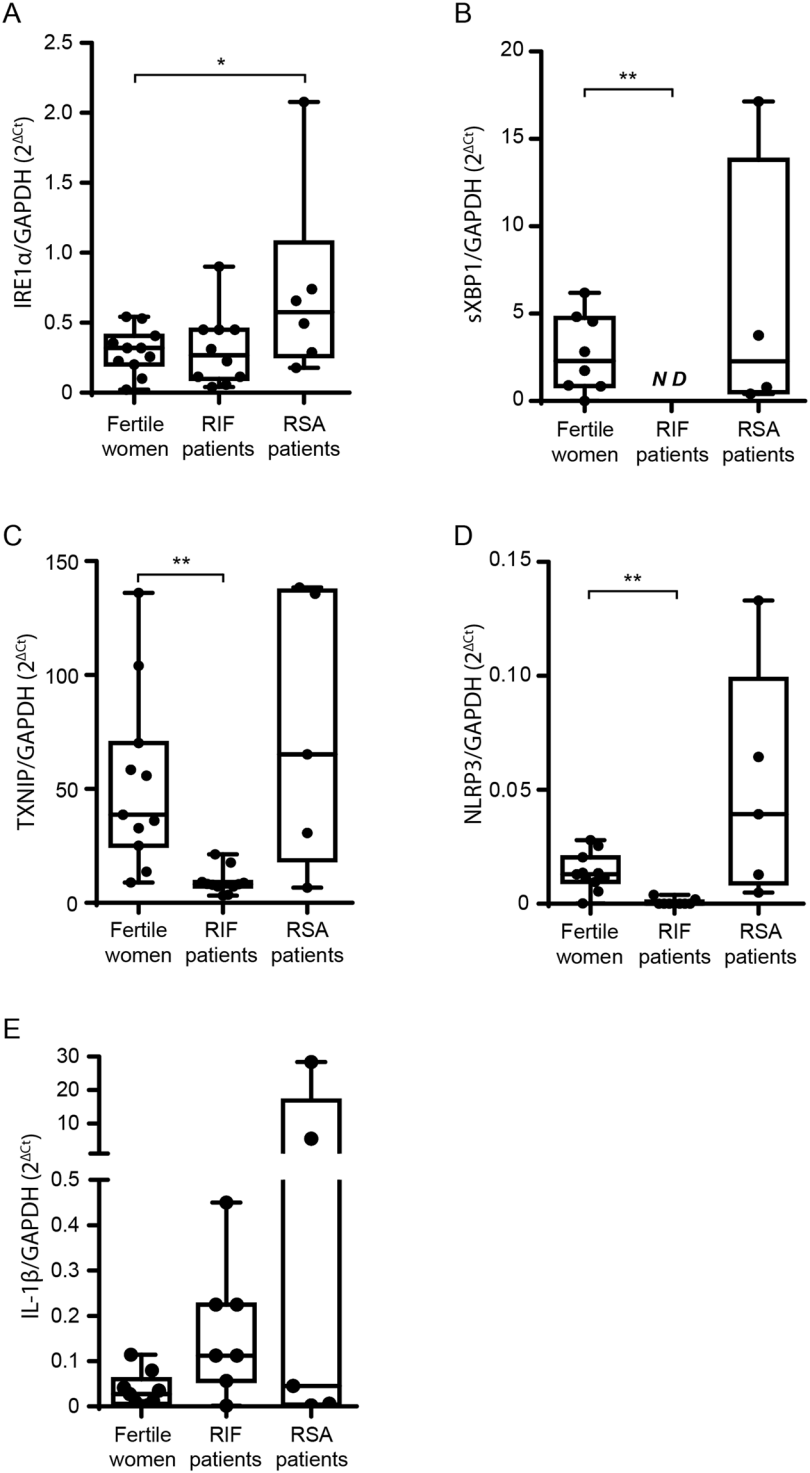
RS/UPR through IRE1α pathway and the induction of IL-1β pathway are differentially expressed in endometrium from patients with recurrent spontaneous abortion (RSA) and from recurrent implantation failures (RIF) in comparison with fertile women. Endometrial biopsies were obtained during the secretory phase of the menstrual cycle from patients with RSA or RIF and from fertile women. Samples were mechanically disrupted, RNA was isolated and cDNA was generated. The RS/UPR pathway, NLRP3 inflammasome and IL-1β expression were analyzed by RT-qPCR. The expression of (**A**) IRE1α, (**B**) sXBP1, (**C**) TXNIP, (**D**) NLRP3 and (**E**) IL-1β were expressed as 2^−Δ^Ct. *p < 0.05, **p < 0.01, nonparametric Kruskal–Wallis test for multiple comparisons with Dunn’s posttest. Data are displayed by a box and whiskers plot, showing minimum, lower quartile, median, upper quartile, and maximum.

Taking into account the previous results, we decided to study if the endometrium samples from patients with recurrent spontaneous abortions (RSA) or with recurrent implantation failures (RIF) display any differences in the induction of the IRE1α - IL-1β -pathway in comparison with endometrium from fertile women. For that purpose, endometrial biopsies were obtained during the secretory phase of the menstrual cycle from the 3 groups under study and gene expression evaluated by RT-qPCR. As Fig. 6 shows, endometrial cells from RSA patients displayed a significant increase of IRE1α expression and a tendency to increase TXNIP and NLRP3 expression in comparison with fertile women. Although no significant differences were observed in IL-1β expression between RSA patients and fertile women, we did observed a positive correlation between IL-1β and IRE1α, TXNIP and NLRP3 expression (r = 0.99 p < 0.01; r = 0.99 p < 0.01; r = 0.91 p < 0.05; Spearman’s correlation coefficient respectively). On the other hand, as Fig. 6 shows, patients with RIF displayed a tendency to increase IL-1β expression, while sXBP1, TXNIP and NLRP3 endometrial expression was significantly lower in comparison with fertile women. These differences are very interesting since RSA patients, even if they have recurrent losses, the blastocysts actually implant, while the endometrium of RIF patients does not allow blastocysts implantation, not even those with high quality. Moreover, these results are also in agreement with those observed in the *in vitro* implantation model (Fig. 5), highlighting that at least low or mild tenors of RS/UPR are required for blastocyst invasion.

## Discussion

Reproductive cycle in humans involves a spontaneous decidualization of the endometrium in an embryo-independent manner^6,19,20^. In the context of fetal-maternal interaction, our findings indicate that decidualization pre-conditions the uterus to an inflammatory response needed for trophoblast cell invasion.

Results presented herein provide experimental evidence that the inflammatory response characteristic of the peri-implantation period might be linked to RS and UPR that endometrial stromal cells undergo during the decidualization process. Our conclusions are based on several observations. First, after decidualization the HESC cells increased the expression of RS-sensors (ATF6, PERK and IRE1α) and UPR markers (sXBP1 and CHOP). However, Dec cells still showed the ability to respond to a strong stressor, suggesting that the physiological differentiation induces low or mild RS/UPR levels. Second, RS/UPR inflammatory pathway activates TXNIP and increases NLRP3 expression, mediating the inflammasome activation. This multiprotein complex is functionally active and induces maturation of Caspase-1. As we expected, Dec cells significantly increased IL-1β protein production accompanied by an increase of *de novo* synthesis. Third, the inhibition of IRE1α-UPR by STF-083010 prevented the increased expression and production of IL-1β in Dec cells, confirming that the IRE1α-pathway is involved in IL-1β production. Forth, the treatment of Dec cells with STF-083010 decreased trophoblast invasion as evaluated in an *in vitro* model of implantation, suggesting that this process is at least partially dependent of the RS/UPR response. Finally, when we studied IRE1α pathway in endometrium samples, we found that RSA patients presented significantly higher expression of IRE1α accompanied of a similar tendency of TXNIP and NLRP3. In contrast, the endometrium samples from RIF patients showed a significant decrease in the UPR mediators TXNIP, sXBP1 and NLRP3 in comparison with fertile women, suggesting that a defective UPR may be involved with implantation issues. These results also suggest that a delicate balance in this pathway is needed, as low levels of the inflammatory response are required to sustain embryo implantation. However, further studies are required to assess the clinical value of our findings.

Here we show for the first time that the physiological RS/UPR during the decidualization process favors a suitable microenvironment for embryo implantation. Previous results from Brosens *et al*. showed that decidualization of HESC cells transfected with pcDNA3/XBP1-luc only marginally enhanced luciferase levels whereas simultaneous HSC70 knockdown elicited a 5-fold induction^21^. These interesting results highlight that the levels of RS/UPR could determine the fate of decidualized cells: on one hand, low to mild tenors of RS/UPR associated with the production of low levels of IL-1β are necessary for blastocyst invasion, as we demonstrated here; and on the other hand, an exacerbate RS/UPR induces the increase of the microtubule-associated protein light chain 3B (LC3B), a marker of autophagic activity^21^. In fact, the down-regulation of the chaperone HSPA8 in decidual cells converts the differentiation-associated UPR into an overt RS response, which in turn compromises secretion of decidual factors essential for placental formation and fetal development.

Cellular differentiation is one of the most well established *in vivo* functions of the UPR^9^. The embryonic lethality of XBP-1 knockout mice derives from failed liver development and IRE1α knockout mice die on embryonic day 12.5 as a result of impaired function of vascular endothelial growth factor (VEGF) and the placental labyrinth layer^22^. Furthermore, similar results have suggested that the placental morphology is impaired and low birth weight is caused by prolonged RS^23^. Therefore, the function of the placenta is impaired either because the RS is lost or generated in an exacerbated manner, highlighting the fine control that exists in this process.

The relevance of the RS as an initiator of the inflammatory response has been addressed. Different pathways, involving NF-κB transcription factor family and MAPKs kinases including JNK, p38, and ERK are involved^12,24^. Particularly, in endothelial cells, IRE1α activation specifically recruits IκB kinase to the ER through the intermediate TRAF2 inducing the production of TNF-α and facilitating stress-induced cell death^25^. However, low RS attenuates TNF-α-induced NF-κB activation reducing the inflammatory response and has other effects such as the upregulation of cell adhesion molecules^26^. How the cells bias towards pro-inflammatory or anti-inflammatory UPR signaling is poorly understood, but the intensity of UPR activation may be a critical mediator of RS and cytokine production. Whether these patterns are specific to certain cell types and operate in decidualized cells *in vivo* remain to be determined.

Regarding the formation of large multiprotein complexes, the inflammasome activation through NLRP3 inducing Caspase-1 maturation and IL-1β secretion is a well known mechanism in response to cellular danger^12,27^. Particularly, in the placenta, Pontillo *et al*. demonstrated that the activation of this pro-inflammatory pathway, NLRP3-inflammasome-IL-1β, was enhanced in response to bacterial LPS in cytotrophoblastic and stromal decidual cells during the first trimester^28^. RS *in vivo* could be induced by both intrinsic and extrinsic factors. The most common physiological intrinsic factor that leads to RS is an increase in protein synthesis, a process which occurs during the differentiation of endometrial stromal cells and is enough to generate a low IL-1β production and contribute to embryo implantation. However, RS contributes to the initiation and progression of numerous diseases in humans due to protein misfolding or defects in UPR pathways induced either by gene mutations or by changes in the environment, such as inflammation, hypoxia or nutrient deprivation^29–31^. Indeed, recently it was identified that the Stromal cell derived factor 2 (SDF2), a protein involved in chaperone network and protein folding, is a regulatory factor by which trophoblast cells can control its survival under RS, which is associated with placental-related diseases^32^.

Regarding the analysis performed in the biopsies, we propose that an altered RS/UPR in endometrium during the implantation window could underlie different receptivity problems, such as those observed in RSA and RIF patients. These alterations might be associated with an altered inflammatory response. Actually, it would be expected that the endometrium of the RSA and RIF patients have different behaviors, since in the case of the first ones, the blastocyst manages to implant and then the abortion occurs between 12^th^ and 14^th^ weeks, while in RIF patients, the endometrium does not allow implantation to occur at all.

In this sense, Galgani *et al*. reported that both sXBP-1 and HSPA5 expression, RS markers, were increased in endometria from RSA patients while RIF patients presented no differences with fertile women^33,34^. In fact, immunohistochemical analysis of the same samples showed that HSPA5 protein levels were higher in the endometria of the RSA compared with the fertile group. Even though this work only presents two markers of RS and UPR, the results obtained are similar to those presented here. In fact, D’Ippolito *et al*. focused on the NALP3 inflammasome activation since is the most versatile inflammasome subtype and the best characterized molecular trigger of IL-1β and IL-18 maturation and release. They found that NALP3 and ASC (apoptosis associated speck-like protein containing a CARD) are increased in endometrial tissues obtained from women with RSA accompanied by an increased activation of Caspase-1 and, as a consequence, an increased secretion of IL-1β and IL-18 compared with the fertile women^35^. The authors postulated that NALP3 could be activated by a range of pathogen-associated molecular patterns (PAMPs), as bacterial muramyl dipeptide, bacterial RNA, or viral RNA, and by DAMPs such as ATP, decreased intracellular potassium concentration, or radical oxygen mediators^35^.

The identification of this deregulated balance of RS/UPR in human endometrium and its potential association with a defective implantation lead the way to the identification of immunomodulatory therapies to improve endometrial receptivity. Further studies including a larger number of women are needed to elucidate the potential role of this sterile inflammatory pathway in the human endometrium *in vivo* and as a possible new promising diagnostic and prognostic biomarker of endometrial receptivity.

## Methods

### Patients

Recurrent implantation failures (RIF) patients were defined as women with a history of two or more cycles of *in vitro* fertilization-embryo transfer (IVF-ET) with one high-quality transferred embryo at least (n = 14).

Recurrent spontaneous abortions (RSA) patients were defined as women with a history of three or more consecutive pregnancy losses before week 12 of gestation after excluding any infectious, endocrine or anatomic disease that might have caused the abortion (n = 9).

Control fertile women were defined as non-pregnant women who had two or more previous normal pregnancies without any miscarriage (n = 17).

The inclusion criteria for all groups were as follows: range age 18–45 years, regular ovulatory cycles (28–31 days), absence of any infectious, endocrine or anatomic disease that might have caused abortion or implantation failure. No significant differences between groups were observed. Table 1 shows patients’ demographic characteristics.

**Table 1:** Patients’ demographic characteristics.

Characteristic	Fertile Women	RSA Patients	RIF Patients
N	17	9	14
Age (y)	38 (31–42)	34 (27–38)	35 (33–39)
Births	2 (1–5)	0	0
Recurrent Spontaneus Abortions	0	4 (3–6)	0
Repeated IVF Failures	0	0	5 (2–9)

Values are shown as median and range.

The Investigation and Ethics Committee from Fertilidad San Isidro center, Buenos Aires, Argentina and from the Universidade Federal de São Paulo (UNIFESP), São Paulo, Brazil, have approved this study. All research was performed in accordance with relevant guidelines/regulations, and the written informed consent was obtained from all participants.

### Cell Lines

Human first trimester trophoblast cell line (Swan-71) and human endometrial stromal cell line (HESC) (a gift by Dr. Gil Mor Medical School, Yale University, USA) were used in these studies. All cells were maintained in DMEM-F12 supplemented with 10% FBS, 50 U/ml penicillin, 50 μg/ml streptomycin and 2 mM glutamine^36,37^. For the different assays, HESC cells were cultured in 24-well plates until they reached 70% confluence.

#### Decidualization

HESC cells were cultured in 24 wells plate with DMEM-F12 10% FBS in the presence of medroxyprogesterone (MPA) (10^−7^ M) and dibutyryl-cAMP (dbcAMP) (2,5 10^−3^ M) for 8 days (Dec), changing half of the culture media and renewing the stimuli every 48 h and then used in the assays described below. In all cases, cells were washed and the differentiation stimuli were removed after decidualization. The decidualization process was confirmed by the evaluation of decidual markers and cell viability, as previously described^18^. Non-decidualized (Non-dec) cells were cultured simultaneously in similar conditions without decidualization stimuli. For certain assays, HESC cells were treated with STF-083010 (10 μg/ml; Sigma-Aldrich) or thapsigargin (1 μg/ml; Sigma-Aldrich) for 4 hours.

### *In vitro* implantation model

#### Blastocyst-like spheroids (BLS) generation

Trophoblast cells from a confluent T25 flask of Swan-71 cells were trypsinized and plated into low attachment P60 plates (Corning Incorporated, Corning, NY, USA). Formation of spheroids was monitored, until they reached a compact spherical morphology (48 h). Cell viability of the spheroids was evaluated by trypan blue staining (viability > 99%).

#### Co-Culture of BLS with HESC cells

HESC cells decidualized and non-decidualized were grown to confluence in 24 wells plate (Greiner Bio-One, Kremsmünster, Austria).The inhibitor STF-083010 was added for 4 h prior to the invasion assay. Swan-71 spheroids were morphologically selected, stained with CFSE (CellTrace CFSE Kit, ThermoFisher Scientific, MA, USA) according providers instructions and ten spheroids per well were transferred using a transfer pipette and a dissecting microscope. The co-culture was maintained with DMEM supplemented with 10% FBS without differentiation stimuli nor STF-083010. All co-cultures were monitored using a fluorescence microscope (Olympus Lifesciences, USA) and microphotographs were taken at 48 h, as we previously determined to be the optimal invasion time^18^. Invasion index was analyzed as morphological change and calculated as “1-minor_axis/mayor_axis” of an ellipse surrounding the BLS as previously reported using ImageJ software (NIH, USA)^18^. For each assay, the average of the invasion index of all the BLS on each well was considered as one single sample and used for the statistical analysis.

### Endometrial Samples Collection

Endometrial samples were taken during the secretory phase of menstrual period (mean 22.56 days) from the first day of the last menstrual period in women with regular cycles. Tissue samples were obtained through the Fertilidad San Isidro center and the Obstetric Outpatient Clinic of UNIFESP under established standard operating procedures. Endometrial biopsies were obtained using a hysteroscopic scissors from women undergoing hysteroscopy control and immediately stored in TRIzol reagent (Life Technologies, USA) at −80 °C up to use. Finally, the samples were disrupted with a Precellys tissue homogenizer and RNA was isolated following manufacturer’s recommendations.

### Real Time PCR

We evaluated IRE1α, PERK, ATF6, sXBP1, CHOP, TXNIP, NLRP3 and IL-1β expression in Non-dec or Dec HESC and endometrial samples from RSA, RIF and fertile women. Total RNA was isolated using Trizol reagent following manufacturer’s recommendations. cDNAs were generated from 1 µg of RNA using a MMLV reverse transcriptase, RNAsin RNAse inhibitor and oligo (dT) kit (Promega, USA) and stored at −20 °C for batch analysis. PCR assays were performed using FastStart SYBR green mastermix (Roche, Germany) following manufacturer’s recommendations on an iQ5 real time PCR (Bio-Rad, USA). Primers sequences and melting temperatures are described in Table 2. Genes expression was quantified relative to the mRNA expression of the endogenous reference gene GAPDH by comparative Ct method, using the 2^−ΔΔ^Ct calculation and expressed as fold increase. In case of endometrial samples, the genes expression evaluated were expressed as 2^−Δ^Ct.

**Table 2:** Primers used in RT-qPCR assays.

Gene	Sense	Primer sequence (5′ → 3′)	Product lenght (bp)	Melting temperature (°C)
GAPDH	F	TGATGACATCAAGAAGGTGGTGAAG	240	62
	R	TCCTTGGAGGCCATGTAGGCCAT		
IRE1α	F	TCACAAAGTGGAAGTACCCG	99	57
	R	AGGCATAGAGGCTGGTAG		
PERK	F	AGACGATGAGACAGAGTTGCG	156	58
	R	TTGCTAAGGCTGGATGACACC		
ATF6	F	TCAAGCACCTGGAGTTCTG	200	57
	R	GTCTCCTTAGCACAGCAATATC		
sXBP1	F	CTGAGTCCGCAGCAGGTG	221	57
	R	GGAGGCTGGTAAGGAACTGG		
CHOP	F	TTAAGTCTAAGGCACTGAGCGTATC	135	55
	R	TGCTTTCAGGTGTGGTGATG		
TXNIP	F	GACCGCCCGAGCCAGCCAAC	156	62
	R	AGAGACAGACACCCGCCCATCAGG		
NLRP3	F	TCAGACAGAGAAGGCAGACC	194	58
	R	GGCATATCACAGTGGGATTCG		
IL-1β	F	TGATGGCTTATTACAGTGGCAATG	140	62
	R	GTAGTGGTGGTCGGAGATTCG		

Oligonucleotide primers were designed using the online tool PrimerBlast (NCBI NIH, USA).

### Flow-cytometry analysis

Non-dec and Dec cells were stained with PE-conjugated mAb anti IL-1β (BD Biosciences, USA) for its intracellular detection or the corresponding isotype control. Cells were fixed and permeabilized using Cytofix/Cytoperm Kit (BD Biosciences, USA) and stained according manufacturer’s instructions. Finally, cells were washed with permeabilization buffer and ten thousand events were acquired in a FACSAria II cytometer. Results were analyzed using FlowJo 7.6 Software and expressed as the mean fluorescence intensity (MFI) of IL-1β.

### FAM-FLICA Caspase-1 Assay

For determination of caspase-1 activation, FLICA reagent was added in Non-dec and Dec cells cultures, according to manufacturer’s instructions (Immunochemistry, Technologies, USA). Briefly, at day 8 of decidualization, cells were washed and incubated with FLICA-probe for 2 h in DMEM-F12 10% FBS. Finally, they were washed thoroughly and ten thousand events were acquired in a FACSAria II cytometer for determination of caspase-1 activation by flow cytometry. Cells without FLICA treatment were used as negative autofluorescence control. Results were expressed as fold increase of the MFI to the Non-dec culture.

### Statistical analysis

Statistical significance of differences was determined by Student’s *t* test in case of pairwise comparisons and ANOVA with Sidak’s posttest in case of multiple comparisons for parametric analysis. For endometrial biopsies analysis, statistical significance was determined using the nonparametric Kruskal–Wallis test for multiple comparisons with Dunn’s posttest. For correlation analysis, nonparametric Spearman’s correlation coefficient was calculated. Statistical significance was defined as p < 0.05, using the GraphPad Prism 6 software (GraphPad, USA).
